# A fatal case of disseminated chronic Q fever: a case report and brief review of the literature

**DOI:** 10.1007/s15010-016-0884-0

**Published:** 2016-03-03

**Authors:** Stephan P. Keijmel, Ruud P. H. Raijmakers, Teske Schoffelen, Maria C. W. Salet, Chantal P. Bleeker-Rovers

**Affiliations:** 1 Division of Infectious Diseases 463, Department of Internal Medicine, Radboud university medical center, P.O. Box 9101, 6500 HB Nijmegen, The Netherlands; 2 Radboud Expert Centre for Q fever, Radboud university medical center, P.O. Box 9101, 6500 HB Nijmegen, The Netherlands; 3 Department of Pathology, Radboud university medical center, P.O. Box 9101, 6500 HB Nijmegen, The Netherlands

**Keywords:** Chronic Q fever, *Coxiella burnetii*, Disseminated infection, Fatal, Q fever

## Abstract

**Background:**

Chronic Q fever is a rare infection, which mainly manifests as endocarditis, infection of vascular prostheses or aortic aneurysms. We present the case of a 74-year-old immunocompromised man with a haematologically disseminated *Coxiella burnetii* infection, which has never been reported before.

**Case report:**

He was diagnosed with a chronic Q fever infection of an aneurysm with an endovascular prosthesis in 2015, but he died despite optimal treatment. Autopsy revealed a disseminated *C. burnetii* infection, confirmed by a positive PCR on samples from several organs. Retrospectively, he already had complaints and signs of inflammation since 2012, for which he had already been admitted in February 2014. At that time, Q fever diagnostics using PCR, complement fixation assay, and enzyme-linked immunosorbent assay on serum were all negative. In retrospect however, retesting available samples from February 2014 using immunofluorescence assay (IFA) already revealed serology compatible with chronic Q fever.

**Conclusion:**

Clinicians should be aware of this silent killer, especially in case of risk factors, and perform an appropriate diagnostic work-up for Q fever including IFA serology and PCR.

## Introduction

Following primary infection with *Coxiella burnetii*, an intracellular Gram-negative coccobacillus, 1–5 % of patients develop chronic Q fever, which is characterized by the persistence of *C. burnetii*. Chronic Q fever mainly manifests as endocarditis, infection of vascular prostheses or aortic aneurysms, or both [1]. Increasingly, other manifestations are reported, such as osteomyelitis, pericarditis, hepatitis, pseudotumor(s) of the lung, chronic pulmonary fibrosis, cerebral venous thrombosis, and musculoskeletal infections [2, 3]. However, there are no reports describing a disseminated chronic Q fever infection with both locoregional and haematogenous seeding of *C. burnetii*. We report a fatal case of a disseminated chronic Q fever infection, confirmed by positive PCR for *C. burnetii* on lung tissue, an endovascular aneurysm repair (EVAR) specimen, a psoas abscess specimen, and ascites from the abdominal right lower quadrant.

## Case report

**Fig. 1:**
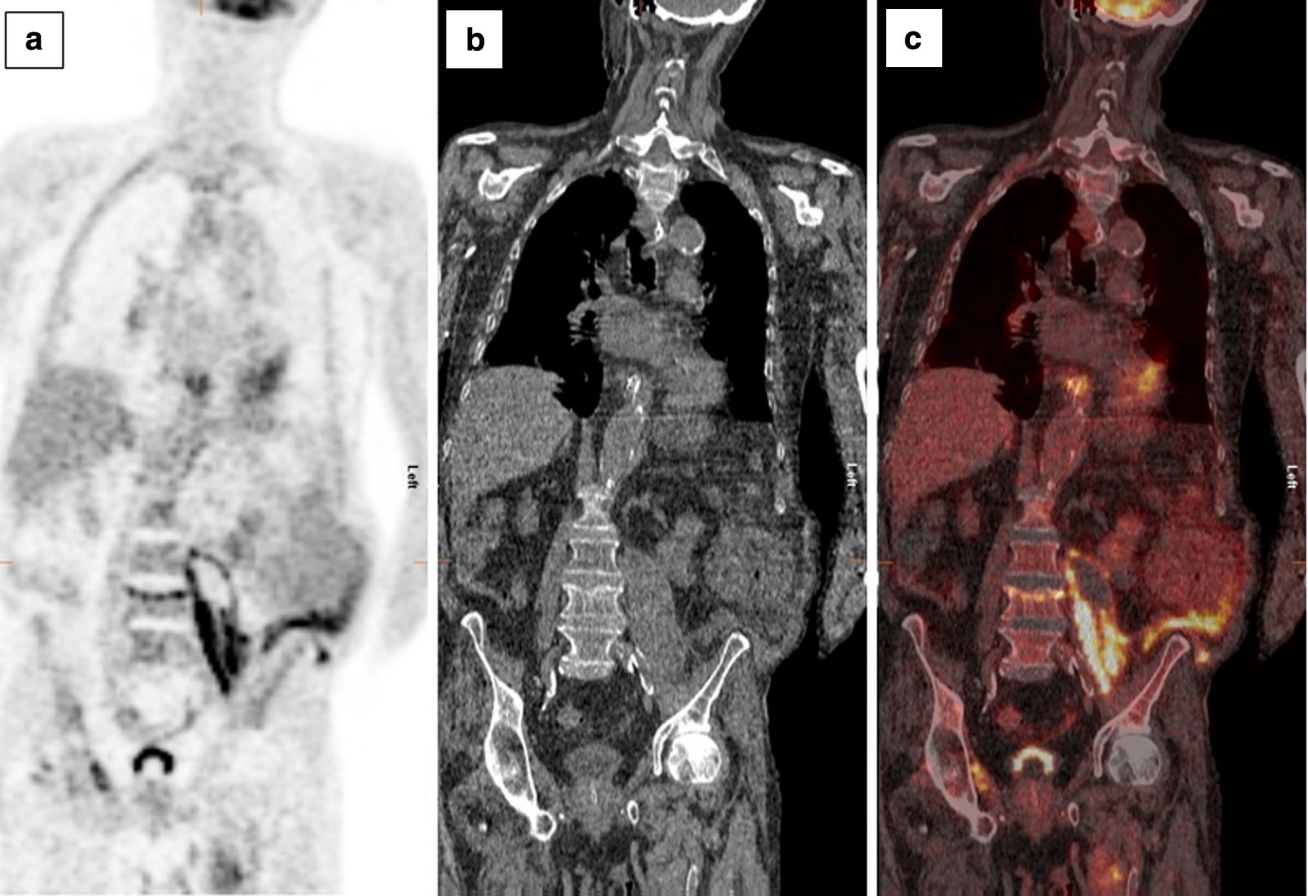
^18^F-fluorodeoxyglucose positron emission tomography (^18^FDG-PET) (**a**), low-dose CT (**b**), and integrated ^18^FDG-PET/CT (**c**) images, demonstrating increased FDG-uptake in the abscess formation in the left iliopsoas muscle, extending into the intervertebral space cranially of L4 and into the adipose tissue reaching the left abdominal wall. The ^18^FDG-PET could not be assessed for disseminated lesions in the brain due to a motion artifact of the head during the procedure

**Fig. 2:**
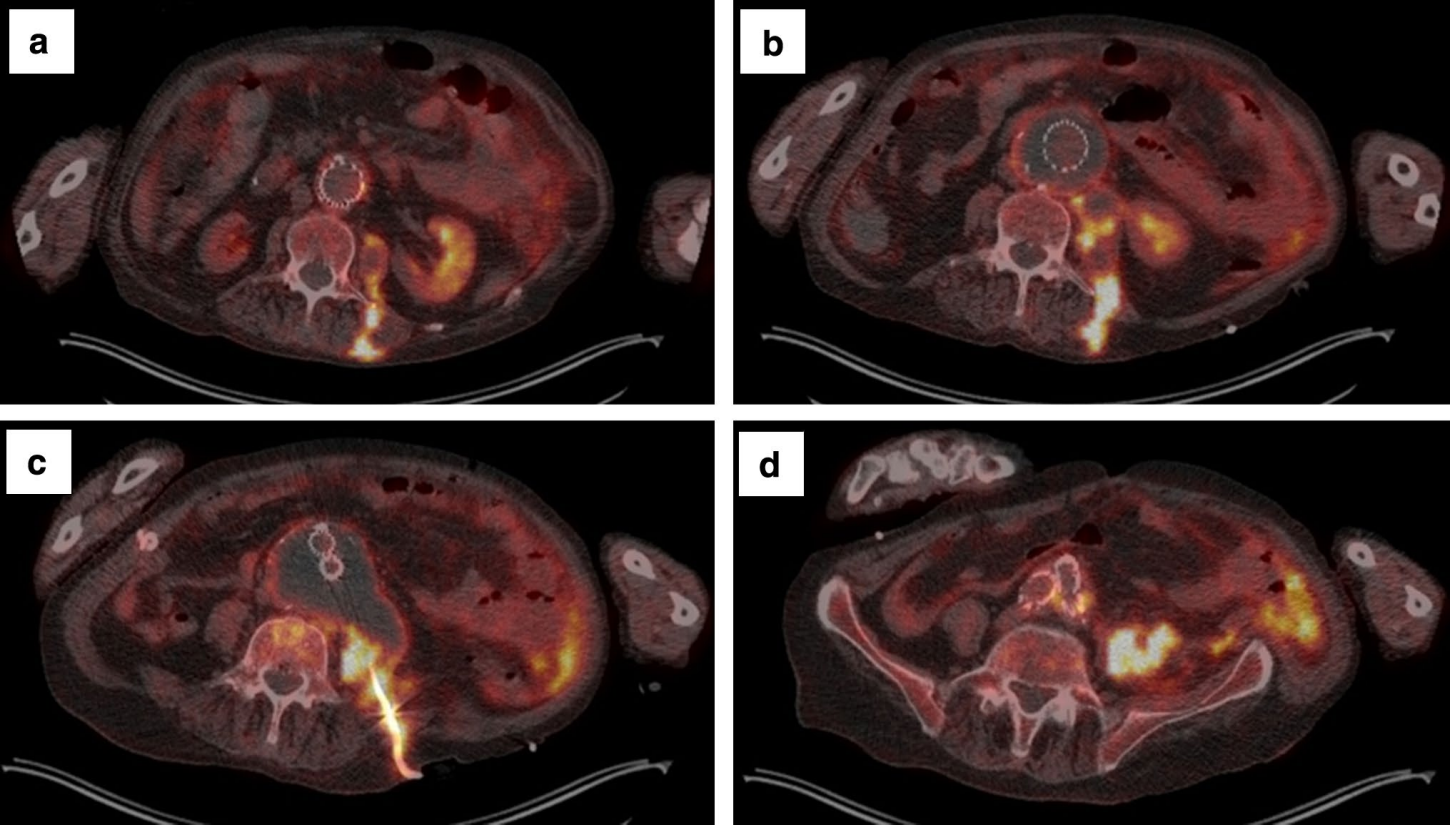
Transversal integrated ^18^F-fluorodeoxyglucose positron emission tomography/low-dose CT (^18^FDG-PET/CT) images, from cranial to caudal, demonstrating: **a** increased FDG-uptake in the left iliopsoas muscle dorsally extending through the musculature of the back, and increased FDG-uptake in the wall of the aortic aneurysm adjacent to the endovascular aneurysm repair (*EVAR*). **b** A per continuitatem infection arising from the abdominal aortic aneurysm (*AAA*), thrombosis of aortic aneurysm and low activity in the cavity of the EVAR resulting from blood flow. The infection extends to the abscess and left iliopsoas muscle. **c** Percutanous drain in situ in the abscess, increased FDG-uptake in the cranial portion of the vertebra, and increased FDG-uptake in adipose tissue of the left abdominal wall in continuitatem with the abscess (not visible at the level of this transversal slice). **d** Increased FDG-uptake in the aortic wall adjacent to the caudal part of the EVAR, and increased FDG-uptake extending into adipose tissue of the left abdominal wall

**Fig. 3:**
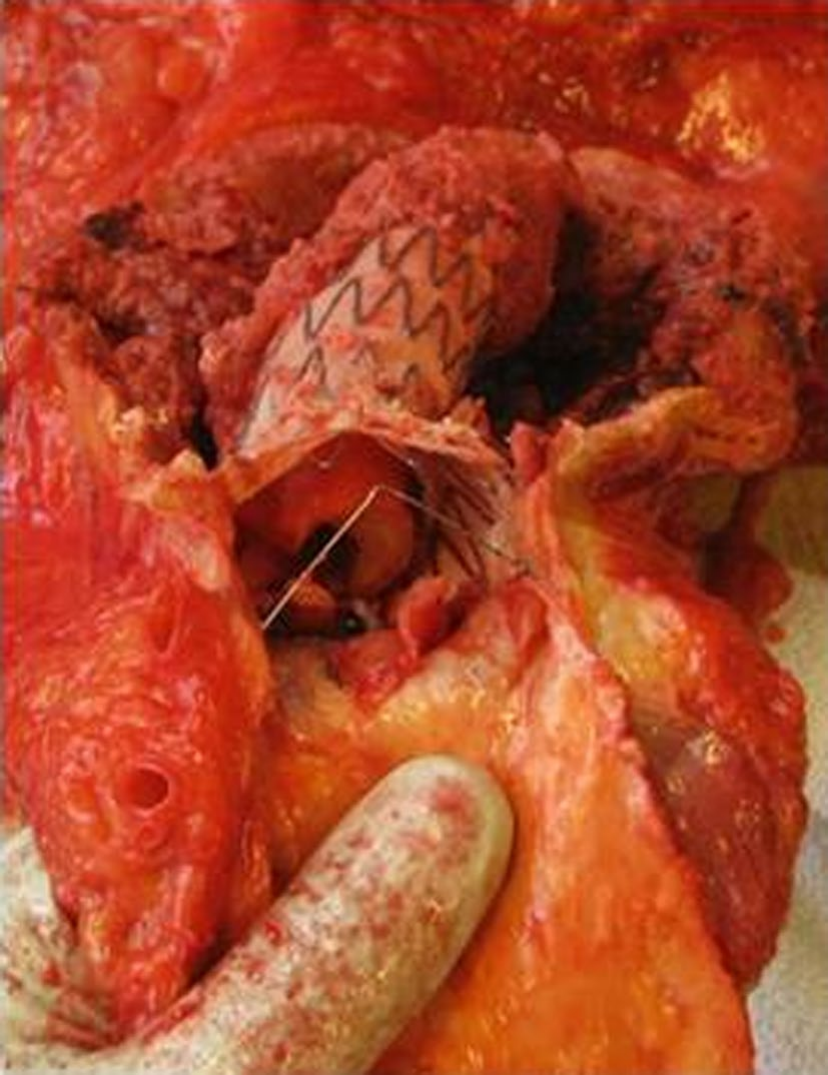
Cranial view, during autopsy, of the abdominal aorta with the endovascular aneurysm repair (*EVAR*) stent-graft. The lumen of the celiac trunk and superior mesenteric artery are visible. Around the EVAR the aneurysmatic plaque inside the dilated vascular wall is still in situ, the material was PCR positive for *C. burnetii*. A fistula from the abdominal aortic aneurysm (*AAA*) to the psoas abscess was present (not visible on picture). Inside the EVAR an intra-prosthetic deposition of amorphous material is visible

A 74-year-old man was admitted to our department in January 2015 with general malaise, weight loss, dyspnoea, abdominal pain and back pain. His history revealed active rheumatoid factor positive rheumatoid arthritis (RA) since 1972, treated with prednisone since January 2000 and abatacept since August 2014, deep venous thrombosis, emphysema, and hypertension. In 2008, an infrarenal abdominal aortic aneurysm (AAA) was diagnosed and treated with an endovascular aneurysm repair (EVAR) in February 2012 after symptomatic presentation. In October 2012, transthoracic echocardiography (TTE) revealed aneurysms of the aortic sinus (44 mm) and ascending aorta (42 mm), without valve abnormalities. In February 2014, increasing back pain and left-sided abdominal pain, without fever, night sweats or weight loss, resulted in admission to the department of Surgery. CT angiography (CTA) showed right renal artery occlusion, and an expanded AAA connecting with a fluid collection around the left iliopsoas muscle. The infectious diseases specialist advised to perform Q fever diagnostics. The PCR (in-house real-time PCR targeting IS1111a), enzyme-linked immunosorbent assay (ELISA, Pan-Bio Pty Ltd., Windsor, QLD, Australia), and complement fixation assay (CFA; Virion-Serion, Würzburg, Germany) on serum were negative. Repetitive TTE in 2014 depicted a stable cardiac condition. On physical examination at presentation in January 2015, he was afebrile with a blood pressure of 184/97 mmHg, with 96 % saturation. Cardiac examination was normal, endocarditis stigmata were absent, as was lymphadenopathy. Pulmonary examination revealed left-sided rales and right-sided crackles. He reported tenderness on palpation of the thoracic spine. Besides a C-reactive protein (CRP) of 67 mg/l (normal range, <5 mg/l) and hemoglobin level of 7.3 mmol/l (normal range, 8.4–10.8 mmol/l), laboratory results were normal. Chest X-ray revealed a recent thoracic spinal fracture, and abdominal ultrasound showed hepatomegaly and a psoas hematoma. CTA showed no leakage of the aortic graft. ^18^F-fluorodeoxyglucose positron emission tomography/low-dose CT (^18^FDG-PET/CT) 3 days later showed a normal FDG distribution in the patients’ head, neck, and brain parenchyma, but a high pulmonary FDG-uptake suggestive for pneumonia, and signs of an infected AAA expanding to the left psoas muscle. CT-guided puncture of the psoas abscess revealed pus, which was PCR positive for *C. burnetii*. Immunofluorescence assay (IFA; Focus Diagnostics Inc., Cypress, CA, USA) showed high anti-*C. burnetii* antibody titres: IgG phase I 1:4096, phase II 1:2048, IgM phase I and II negative. Serum PCR remained negative. Chronic Q fever was diagnosed and treatment with doxycycline 200 mg/day and hydroxychloroquine 600 mg/day was initiated. Prednisone (5 mg/day) was continued, but abatacept was stopped and the abscess was drained percutaneously. Shortly after being discharged, he was readmitted because of collapse, confusion, and increasing back pain. CT showed a new thoracic aortic aneurysm (52 mm) and an expanded multiloculated psoas abscess, which again was drained percutaneously. In the absence of a clinical response, moxifloxacin 400 mg/day was added, but had to be stopped due to a markedly prolonged QTc-interval. Despite several drains in the multiloculated abscess, CRP increased to 261 mg/l and he developed a fever. His hospital stay was complicated by two episodes of presumed hospital-acquired pneumonia (for which he received piperacillin/tazobactam), acute decompensated heart failure, respiratory failure presumably due to an aspiration pneumonia, and sepsis, for which he was temporarily transferred to the intensive care unit twice. Furthermore, he developed a gastroparesis, acute progressive renal insufficiency and a delirium. A new ^18^FDG-PET/CT (Figs. 1, 2) showed increased FDG-uptake extending into the vertebrae and high FDG-uptake in his spleen suggestive for satellite infection. Despite treatment with adequate doxycycline levels, the patient died 4 months after presentation. Autopsy was performed, macroscopically showing inflamed tissue around the EVAR (Fig. 3) with fistulas to the iliopsoas muscle in continuation with the spine with softened vertebrae. Microscopy yielded a chronic granulomatous necrotizing inflammation of the aortic vascular wall around the EVAR, fully necrotic iliopsoas muscle and surrounding area, and a hypertrophic cardiomyopathy. Necrotizing granulomas were found in both lungs, being PCR positive for *C. burnetii*, as were EVAR specimens, pus from the psoas abscess and ascites from the abdominal right lower quadrant around the appendix. Cultures for *C. burnetii* remained negative. Post-mortem examination of the brain was not performed. Retrospectively, IFA was performed on stored serum from February 2014, already showing an IgG phase I 1:4096, IgG phase II 1:2048, with negative IgM phase I and phase II, suggestive for chronic Q fever. Retesting the stored serum with CFA and ELISA confirmed the previously found negative results.

## Discussion

We describe an immunocompromised patient with a widely disseminated chronic Q fever infection with infectious foci in the EVAR and surrounding AAA, both lungs, iliopsoas muscle, spine, spleen, and in ascites from the abdominal right lower quadrant. To our knowledge, such an extensive *C. burnetii* infection has not been described before. Rare complications, e.g., osteomyelitis [2], periaortic adenopathy, aortaduodenal fistula, psoas abscesses [4, 5], and fistula to the groin [6], have been described as part of locoregional spreading of *C. burnetii*. Such locoregional expansion is probably the result of a contiguous infected vascular aneurysm. In our patient, however, besides locoregional spreading, haematogenous seeding of *C. burnetii* is likely because of signs of metastatic infection in the spleen and the presence of *C. burnetii* DNA and granulomatous inflammation in lung tissue. Haematogenous spread can also result in hepatic abscesses, described in one patient with both splenic and hepatic abscesses [3]. However, this occurred during an acute *C. burnetii* infection, instead of chronic Q fever as in our case, with complete resolution of symptoms and abscesses after 21 days of doxycycline.

Probably the immunocompromised state of the patient (due to the use of abatacept and prednisone) contributed to the widespread infection. A disseminated Q fever infection with acute endocarditis in experimentally infected immunocompromised mice 10 days after intraperitoneal inoculation of *C. burnetii* has been described, showing microabscesses, granulomas, and microthrombi in spleen, liver, myocardium and bone marrow [7]. Such a disseminated infection was also found in immunocompetent mice [8]. However, these self-limiting systemic infections were found after intraperitoneally induced acute infection, with characteristic histopathological changes only in the acute setting, whereas persistent infection was found only in the kidneys of a single immunocompromised animal [7]. Abatacept treatment, so far, has not been complicated by many opportunistic or serious infections, in contrast to anti-TNF treatment [9]. However, based on a small number of RA patients, the use of TNF blockers was not associated with increased risk of chronic Q fever, in contrast to corticosteroid use [10], which our patient also used. In addition, it was suggested that RA and its treatment, either with or without anti-TNF, may be considered as a risk factor for chronic Q fever development, and it was advised to monitor RA patients carefully in case of *C. burnetii* infection [10]. The role of abatacept in the dissemination of *C. burnetii* in our patient remains unresolved. Abatacept, inhibiting T cell activation by preventing co-stimulatory interaction between CD80/CD86 and CD28, did not prevent formation of *C. burnetii*-positive granulomata, corresponding with previous findings in *C. burnetii*-infected CD28-deficient mice, in which granuloma formation was also not affected [11]. Interestingly, in these CD28-deficient mice, the *C. burnetii* burden in infected tissue was decreased, suggesting that costimulation of CD28 increases *C. burnetii* replication, implicating a favourable effect of abatacept. Although abatacept was stopped, prednisone was continued during the course of disease because of the long-term use with subsequent hypothalamic–pituitary–adrenal axis suppression. In addition, the patient needed steroid stress dosing due to several complications. However, despite the continuation of prednisone in this specific case, physicians should always consider stopping immunosuppressive therapy while treating chronic Q fever. Another explanation for the widespread infection might be *C. burnetii* resistance to doxycycline, as doxycycline resistant isolates do exist [12, 13]. However, this does not appear to be a common occurrence [14], and it is more likely that the patient died due to an already widely disseminated Q fever infection at the time doxycycline and hydroxychloroquine were initiated, while the immunosuppressive therapy favoured the expansion of the infection.

Diagnosing chronic Q fever is challenging, and often delayed because of the lack of recognition by physicians, mainly due to non-specific symptoms and unfamiliarity with chronic Q fever. However, early diagnosis has major implications, as chronic Q fever causes high morbidity and mortality [1]. Eventually, the indication to test for Q fever was recognized in this case, but retrospectively the patient already reported general malaise for years, chronic chest pain and left flank pain ever since the EVAR procedure. Furthermore, he already had an elevated CRP whilst consulting the cardiologist, pulmonologist and rheumatologist in the years before presentation, who related this to his active RA and intercurrent problems. Our patient lived in an area in the Netherlands with the highest incidence of Q fever during the large Q fever outbreak from 2007 to 2010 [15, 16], and inhalation of contaminated aerosols was probably the route of initial infection [17]. In Q fever endemic areas or in the years after outbreaks, physicians should stay alert on signs and symptoms suggestive for chronic Q fever, especially in case of risk factors, also in the absence of a known acute Q fever episode. Well-known risk factors for developing chronic Q fever include vascular grafts and aneurysms, cardiac valve prosthesis or valvulopathy, and immunosuppresion [18]. Despite the fact that EVAR specimens appeared to be PCR positive for *C. burnetii*, the EVAR could not be revised in this case. The main reason for the decision to abstain from surgical intervention was the already expanded infection, and the patients’ deteriorating physical condition. However, in case of a chronic Q fever infection of a vascular prosthesis, surgical interventions can lead to a better outcome and should always be considered [2, 19]. Our case further emphasizes the need for using IFA to screen for chronic Q fever, as CFA and ELISA have limited sensitivity. Also, this case illustrates that PCR alone is insufficient to rule out chronic Q fever due to the low sensitivity in blood specimens [1].

## Conclusion

In conclusion, we report a fatal case of an immunocompromised patient with a confirmed disseminated chronic Q fever infection, underlining the severity of this disease and the diversity of signs and symptoms that may occur, and highlighting the need for increased awareness and recognition by physicians especially in case of risk factors. Furthermore, we advocate performing an adequate diagnostic work-up using at least IFA serology and PCR for screening for chronic Q fever.

## List of abbreviations

*C. burnetii*: *Coxiella burnetii*
EVAR: Endovascular aneurysm repair
RA: Rheumatoid arthritis
AAA: Abdominal aortic aneurysm
TTE: Transthoracic echocardiography
CTA: CT angiography
ELISA: Enzyme-linked immunosorbent assay
CFA: Complement fixation assay
CRP: C-reactive protein
^18^FDG-PET/CT: ^18^F-fluorodeoxyglucose positron emission tomography/low-dose CT
IFA: Immunofluorescence assay
